# Effects of Hand-Rearing and Group Size on Chimpanzee (*Pan troglodytes*) Social Competence in Captivity

**DOI:** 10.3390/ani16020250

**Published:** 2026-01-14

**Authors:** Lindsay E. Murray

**Affiliations:** Division of Psychology, University of Chester, Chester CH1 4BJ, UK; l.murray@chester.ac.uk

**Keywords:** chimpanzee, group size, rearing, behavior, social competence

## Abstract

The behavior of individual animals reflects both internal states and external context. For captive animals, their early life experiences can influence later adjustment, particularly their social competence. In addition, the way in which they are housed and their current social grouping context are important factors affecting the expression of social skills. Here, the social behavior of 39 chimpanzees (*Pan troglodytes*) was observed to assess whether differences existed due to how the apes were reared and grouped. Behaviors recorded related specifically to five main components: Sociability, Grooming, Play, Responsibility and Aggression. Chimpanzees in larger groups groomed and played more, while those housed in pairs or trios displayed more and spent more time alone. Mother-reared chimpanzees took more responsibility for soliciting grooming, and interactions between grouping and rearing on chimpanzee agonistic display rates highlighted how both hand- and mother-reared chimpanzees display more if housed in small groups rather than large. Implications for the optimal management and welfare of this highly intelligent and social species are discussed in light of the importance of early life experiences in modulating the impact of current social environment on chimpanzee social profiles.

## Simple Summary

Early life experience can impact later adjustment, particularly social competence. For zoo animals, current social grouping also influences behavior. Here, 39 chimpanzees were observed to explore the influence of rearing and grouping on social behavior. Chimpanzees in larger groups groomed and played more, while those housed in pairs or trios displayed more and spent more time alone. Mother-reared chimpanzees took more responsibility for soliciting grooming, and interactions between grouping and rearing on chimpanzee agonistic display rates highlighted how both hand- and mother-reared chimpanzees display more if housed in small groups rather than large. Recommendations for the housing and keeping of this highly intelligent and social species are discussed in light of the importance of early life experiences in modulating the impact of current social environment on chimpanzee social profiles.

## 1. Introduction

The behavior of individual animals reflects both internal states and external context [1]. For captive animals, their early experiences contribute to later behavior which can also be shaped by the way in which they are housed. When highly intelligent and social animals such as chimpanzees (*Pan troglodytes*) are kept in captivity, it is essential that their needs are met in order to provide good welfare. Research has shown that behaviors classed as abnormal, including coprophagy and regurgitation/reingestion, are present in all captive chimpanzee groups regardless of size or composition [2], although higher rates of these behaviors are reported for chimpanzees living in pairs compared to small groups of three to seven individuals [3], and apathy and rocking are more prevalent in hand-reared chimpanzees [4]. Such findings can be related to social deprivation in early life [5]. In such a cognitively and socially sophisticated species, the company of conspecifics is paramount [6]. Yet, there is little research on how much the group size of captive chimpanzees matters. In the US, both the National Institutes of Health [7] and the Association of Zoos and Aquariums [8] recommend a group size of at least seven or eight individuals, preferably mimicking the wild social structure of multi-male, multi-female, age-diverse composition; while European guidelines do not specify a particular number of individuals but recommend keeping more than one adult male with several adult females and their offspring [9]. However, it has been argued that trying to recreate such natural conditions may not be necessary because captive chimpanzees are not faced with the same pressures as their wild counterparts and zoos need to be able to consider other issues such as allowing sufficient physical space per individual and mitigating potential intra-group aggression [10]. Nevertheless, these authors did find that chimpanzees living in groups of at least seven displayed the most locomotion and the most affiliation. The vast body of welfare research also points to the size and features of enclosures being important [see 6 for review], yet a systematic way for practitioners to quantitatively rank enclosure quality has only recently emerged [11]. Regardless of other worthy features in a captive chimpanzee’s environment, the presence of other chimpanzees should be the priority [12].

The fission-fusion social system of chimpanzees in the wild is well documented [13], with individuals moving around their home ranges in dynamic and fluctuating temporary associations, parties or sub-groups, with a median party size of about four individuals [14]. Females typically emigrate from their natal communities, whereas males only rarely move [15,16]. This norm of free-living chimpanzee social structure contrasts strongly with the conditions of captivity, where all members of the group are always present.

The compatibility of different individuals formed into captive groups can vary greatly, and assessment of personality should be taken into consideration. The field of animal personality has burgeoned over recent years and, although still contested in terms of definitions and methods, can be largely regarded as a temporally stable and situationally consistent way of behaving; see, e.g., [17,18]. Personality traits of individual chimpanzees, for example, are found to relate to observed behavior over a period of 25 years [19]. According to evolutionary personality psychology, observed behavior is the result of interaction between evolved psychological mechanisms and the environmental conditions which activate them differentially among individuals [20,21]. Behavior cannot be seen as reflecting purely environmental context; it has to involve organismic mechanisms; in other words, information processing devices have adapted over time to consider sensory input and deploy appropriate overt responses. Thus, while these psychological mechanisms are consistent over time, overt behavior is context dependent. Situational factors such as the way in which animals are grouped and housed therefore have an important role to play in the determination of behavior. The expression of personality in chimpanzees also varies depending on whether they are housed in a group, individually or paired; for example, chimpanzees living in large groups rate higher on items including playful, gentle, protective, intelligent, sociable and curious [22]. Here, I turn to the examination of the effects of grouping and rearing on indices of social competence.

Another important aspect to consider is the animal’s background. How an animal experiences its early social environment can have profound effects on the ontogeny of social competence later in life [23,24]. Social competence, referring to the ability to optimally express social behavior [25], requires an animal to be able to process social information and respond appropriately. The environment in which chimpanzees are reared strongly impacts their behavior as an adult; see, e.g., [26,27]. Wild-born male chimpanzees groom less than captive-born individuals and this distinction took precedence over any shorter-term changes in behavior in response to changes in group composition [28]. Similarly, such changes to current group composition, including the introduction of new members, are also found not to disturb the stability and cohesiveness of the group social network [29]. Such findings may attest to the benefits of group-living in terms of carrying individuals through significant life events. Nevertheless, whether or not captive chimpanzees were hand-reared negatively affects their health, stress, and subjective wellbeing [30] and affects the expression of their personality, with hand-reared individuals rated higher on Effective, Protective and Eccentric [22]. Interestingly, Clay et al. [30] did not find that the increased human exposure of hand-rearing affected how chimpanzees behaved socially with each other, and Martin [31] found no differences in personality traits in differentially reared chimpanzees, but did find that raters found it harder to rate non-mother-reared individuals, possibly due to a less consistent presentation of personality traits, or to the different relationships these individuals had with the human keepers.

The importance of maternal care in the development of young has been demonstrated across a wide range of taxa; see, e.g., [32,33]. The presence of the mother chimpanzee in rearing and teaching her young has been emphasized continually in primatology; see, e.g., [13,34]. As with humans, the mother is the first playmate and, from play, an entire developmental trajectory is set [35]. Bonds between mothers and offspring extend beyond weaning [36] and maternal deprivation is likely to result in negative outcomes including anxiety and depression [37] and difficulties with social integration [38].

It is likely also that factors including grouping and rearing could interact to affect individual chimpanzees in different ways; for example, differences in the personality traits of chimpanzees that were mother-reared compared to hand-reared were more pronounced when those individuals were housed in large groups [22]. Other individual differences such as age and sex are also important and may moderate the effects of rearing [39]; for example, the link between hand-rearing and eccentricity was more pronounced in female chimpanzees [22].

The development of social competence is poorly understood and most research has experimentally manipulated the early environment of species such as fish [23] and birds [24]. This study compares the behaviors of chimpanzees who have experienced hand-rearing compared to being reared by their mothers. It also takes into account the current social structure of the individual chimpanzees, specifically comparing those residing in groups versus in pairs or trios. The behaviors reported here include associations and responsibility for proximity, grooming, aggression and play. I predicted that there would be differences in behaviors of hand-reared and mother-reared chimpanzees, and between those housed in groups compared to pairs or trios. Intuitively, it would be expected that mother-reared and group-living chimpanzees would exhibit higher rates of sociability compared to those who had experienced hand-rearing and those not housed as part of a group. It is also predicted that hand-reared chimpanzees would show more sociable behaviors, indicating greater social competence, when residing in a large group compared to their counterparts in pairs or trios.

## 2. Materials and Methods

### 2.1. Subjects and Study Sites

Thirty-nine chimpanzees were studied for over 1000 h in 1992 in three zoological collections in England (Table 1).

Zoo A housed a multi-male group with four generations of individuals. Their outside enclosure was a 2000 m^2^ grass island surrounded by a water moat. The indoor area, 13 m wide and 12 m at its highest point, contained a large tubular steel climbing frame, wooden platforms, ropes, tires and a smaller water moat. The chimpanzees were fed daily at 0800 h and 1700 h in the off-exhibit area and at 1415 h in the outdoor enclosure. Those individuals who experienced hand-rearing included wild-caught individuals brought to the zoo within their first five years, and individual chimpanzees who received supplemental shorter-term fostering by zookeepers when their mother lacked experience.

The group at Zoo B had an outdoor area comprising two 120 m^2^ rectangular enclosures connected by a weld mesh passage. Roof areas were covered with a ‘space-frame’ climbing apparatus from which ropes were hung. The two indoor areas contained ropes and climbing frames. The chimpanzees were fed at 0900 h in the outdoor enclosures and at 1200 h and 1530 h in the indoor areas. Hand-reared individual chimpanzees at this zoo received supplemental shorter-term fostering by zookeepers when their mother lacked experience.

Zoo C housed its chimpanzees in nine different groups: seven adult chimpanzees were housed in an indoor enclosure with access to an outside dry-moat grass enclosure. Thirteen adult chimpanzees were housed in five groups of two or three animals, housed in partitioned indoor areas with adjoining partitioned outdoor enclosures. Hand-reared individual chimpanzees at this zoo received supplemental shorter-term fostering by zookeepers when their mother lacked experience. It is important to note that these data were collected at a time that coincided with the onset of the broader movement to reduce artificial rearing. Distribution of subjects, by age and sex, into grouping categories is shown in Table 2.

### 2.2. Data Collection and Analysis

Data were collected by a single observer using continuous recording of focal subjects for fifteen minute periods [40]. Individuals were observed in a counter-balanced order and observations covered all times of the day during normal zoo opening times, between 1000 h and 1600 h. The observer stood in the viewing areas of the indoor and outdoor enclosures. Social behaviors for each individual were calculated as frequencies and durations of activity. Association time per dyad, based on recording proximity partners within two arms’ reach, was calculated as percentage of time spent together, and the proximity index for each dyad was calculated, based on the initiator and terminator of each interaction, using the Hinde and Atkinson index [41]. Reliability of behavior codings was established by using an inter-observer reliability check between the observer and another independent observer using Cohen’s Kappa. The behavioral definitions and indexes are shown in Table 3.

A principal component analysis (PCA) was carried out on the behavioral measures. With regard to the Kaiser criterion and examination of the scree plot, five components were extracted from the PCA with varimax rotation, accounting for 75.92% of the variance in behavior codings for chimpanzees (Table 4). Although the Kaiser–Meyer–Olkin measure of sampling adequacy was on the lower side at 0.466, Bartlett’s test of sphericity (χ^2^_105_ = 458.17, *p* < 0.001), indicated that the data were appropriate for PCA. Scores were computed for each chimpanzee on each component using the regression method [42,43]; these scores have zero as the average and show levels of deviation in either direction (above or below average). They retain the important psychometric aspects of factor analysis, using maximum separation between constructs, and different weightings for each item, so that items with higher or lower loadings get more or less weighting, respectively. This permitted analyses of the effects of grouping and rearing on both component scores and discrete behavior measures.

I used mixed-effects linear regression models to quantify the effect of rearing and grouping on the behavioral measures. Each model included rearing and grouping as fixed effects and subject as a random effect to control for the threat to independence of multiple (though different) measures per subject. Where a significant association between the PC score (e.g., Grooming) and grouping or rearing was found, I ran an additional mixed-effect linear regression model which partitioned the model by discrete behavioral measure (e.g., grooming given, grooming received) in order to better understand the specific behaviors driving the overall association. All analyses were conducted using SPSS v.29. Appropriateness of models was assessed by QQ plot visual examination of dispersion of residuals, all of which were found to be acceptable, and by checking variance inflation factors (VIF), all of which were <2, and Cook’s distance, all of which were <1. Final models were selected using Akaike’s Information Criterion (AIC) values.

Ethical Note: Data collection was approved by the University of Cambridge Ethics Committee and the three zoos. There was no manipulation of the animals or their environment, and animals were observed only during their normal display hours at the zoo.

## 3. Results

Inter-observer reliability was checked between the observer and an independent observer, yielding a Cohen’s Kappa of 0.83. Table 4 shows the PCA loadings of the behavior codings on Components 1 to 5.

Table 5 shows the descriptive statistics for chimpanzees’ scores on the behavior components and discrete behavior codings.

### 3.1. Grooming

There was a significant positive association between grouping and Grooming score (Table 6), indicating that chimpanzees in larger groups had higher Grooming scores (M: 0.71, SD: 0.23) than those in smaller groups (M: −0.51, SE: 0.35).

Table 7 presents the data for the behavioral measures influenced by grouping, which will be discussed under each of the relevant component sections. 

Type III Tests of Fixed Effects indicated a significant overall effect of Grouping on median grooming time (*F*(35) = 4.46, *p* = 0.042) and median time being groomed (*F*(35) = 9.62, *p* = 0.004). While main parameter estimates for Grouping did not show clear differences (Table 7), post hoc pairwise comparisons using estimated marginal means revealed that chimpanzees in small groups spent a significantly greater median time grooming (M: 61.53, SE: 13.59) and being groomed (M: 80.23, SE: 15.09) than those in large groups (M: 27.35, SE: 8.80 and M: 24.49, SE: 9.77, respectively). Likewise, these high proportions of time spent grooming involved a higher percentage of significant partners groomed which differed between groups (*F*(35) = 4.21, *p* = 0.048), and a higher percentage of groomers which differed between groups (*F*(35) = 4.20, *p* = 0.048) (although see caveat under sociability below), meaning that individuals in small groups had a higher percentage of significant partners groomed (M: 64.77, SE: 10.59) and significant groomers (M: 64.77, SE: 10.70) compared to chimpanzees in larger groups (M: 38.89, SE:6.86 and M: 38.64, SE: 6.93, respectively, Table 7). For rates of grooming and being groomed, these patterns were repeated, but in the opposite direction. There were significant overall intercept effects for rate of grooming (*F*(35) = 38.79, *p* < 0.001) and rate being groomed (*F*(35) = 32.59, *p* < 0.001). Chimpanzees in large groups had higher rates of grooming (M: 14.30, SE: 1.90) and being groomed (M: 13.51, SE: 1.92) than those in small groups (M: 7.52, SE: 2.94 and M: 6.63, SE: 2.96 respectively).

### 3.2. Play

Type III Tests of Fixed Effects indicated a significant overall effect of Grouping on Play scores (*F*(35) = 8.73, *p* = 0.006). While main parameter estimates for Grouping did not show clear differences (Table 8), post hoc pairwise comparisons using estimated marginal means revealed that chimpanzees in large groups had significantly higher play scores (M: −0.22, SE: 0.15) than those in small groups (M: −1.02, SE: 0.23).

For Median play duration, there was an overall significant intercept effect (*F*(35) = 10.55, *p* = 0.003) and chimpanzees in large groups had higher medians (M: 15.51, SE: 2.82) than those in small groups (M: 1.36, SE: 4.36). Similarly, for Rate of play, the intercept was significant (*F*(35) = 7.42, *p* = 0.01) and chimpanzees in large groups had higher rates of play (M: 4.61, SE: 0.95) than those in small groups (M: 0.14, SE: 1.47). For percentage of significant play partners, the intercept was also significant (*F*(35) = 6.51, *p* = 0.02) and chimpanzees in large groups had significantly more play partners (M: 11.51, SE: 2.45) than those in small groups (M: −1.47 (E-15), SE: 3.79). The intercept was also significant for Responsibility for play (*F*(35) = 5.01, *p* = 0.032) and chimpanzees in large groups took significantly more responsibility for initiating play (M: 0.65, SE: 0.16) than those in small groups (M: 2.08 (E-17), SE: 0.25).

### 3.3. Sociability

Grouping was also positively associated with Sociability scores (Table 9), indicating that chimpanzees in smaller groups had higher Sociability scores (M: 1.24, SE: 0.35) than those in larger groups (M: −0.31, SE: 0.23). This finding, and those of the discrete measures reflecting sociability, need to be interpreted carefully.

There was a significant positive association between grouping and median association times, indicating that chimpanzees in smaller groups had higher association times (M: 41.25, SE: 3.23) than those in larger groups (M: 12.96, SE: 2.09, Table 5). Likewise, these high proportions of association time were spent with a higher percentage of significant partners. However, it should be noted that this reflects those in pairs or trios having to necessarily spend a greater proportion of their time with the constrained number of partners available, meaning that individuals in small groups had a higher percentage of significant partners (M: 80.68, SE: 7.45) compared to chimpanzees in larger groups (M: 49.19, SE:4.82, Table 7).

Type III Tests of Fixed Effects indicated a significant overall effect of Grouping on percentage of time spent alone (*F*(35) = 8.63, *p* = 0.006). While main parameter estimates for Grouping did not show clear differences (Table 7), post hoc pairwise comparisons using estimated marginal means revealed that chimpanzees in small groups spent a significantly greater percentage of time alone (M: 46.36, SE: 6.24) than those in large groups (M: 24.54, SE: 4.04).

### 3.4. Aggression

No significant effects of grouping or rearing were found on Aggression scores (Table 10). Although the intercept suggests that there is likely little meaningful variation in Aggression scores, when discrete scores were examined, some interesting findings were uncovered.

Table 11 presents the data for the behavioral measures influenced by rearing referred to above.

**Figure 1:**
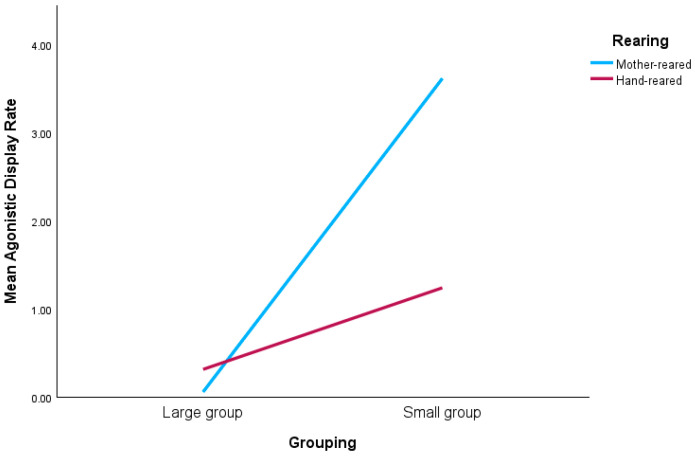
Interaction showing that mother- and hand-reared chimpanzees have higher agonistic display rates (p/hr) when housed in small groups compared to large groups.

Both grouping and rearing affected chimpanzee agonistic display rates; in addition, they interacted to modulate this behavioral outcome (Table 7 and Table 11). Individuals in small groups displayed more (M: 2.43, SE: 0.43) than those in larger groups (M: 0.19, SE: 0.28), and mother-reared chimpanzees displayed more (M: 1.84, SE: 0.47) than hand-reared individuals (M: 0.78, SE: 0.21). Interactions between grouping and rearing on agonistic display rate show that mother- and hand-reared chimpanzees displayed more when they were housed in small groups compared to when housed in larger groups (Figure 1).

### 3.5. Responsibility

No significant main effects of grouping or rearing were found on Responsibility scores (Table 12).

There was, however, a significant positive association between rearing and Responsibility as groomee (Table 11) indicating that mother-reared chimpanzees took more responsibility to solicit grooming from others (M: 1.09, SE: 0.21) than did hand-reared individuals (M: 0.54, SE: 0.09). In addition, there was a trend towards an interaction between rearing and grouping (*p* = 0.055) whereby mother-reared chimpanzees in small groups took more responsibility than their peers in large groups and hand-reared individuals took more responsibility in large groups.

## 4. Discussion

I found clear differences in chimpanzee social behavior reflecting how individuals were grouped and reared, thus giving support to the hypotheses. Chimpanzees residing in larger groups had higher Grooming and Play scores, gave and received more frequent grooming, played more, and took more responsibility for play. Those in smaller groups groomed for longer durations with fewer partners, had higher Sociability scores due to higher median association times spread amongst a higher percentage of significant partners, spent more time alone and performed more agonistic displays. Like some, but not all, previous studies [23,24], the chimpanzees in this study showed differences in social competence related to their rearing. Mother-reared chimpanzees took more responsibility in soliciting grooming than hand-reared apes.

In addition, there were interactions between grouping and rearing on chimpanzee social behavior. Individuals in small groups engaged in agonistic displays more frequently than those in larger groups and mother-reared chimpanzees displayed more than hand-reared individuals. Interactions between grouping and rearing on agonistic display rate show specifically that mother- and hand-reared chimpanzees displayed more when they were housed in small groups compared to when housed in larger groups.

Chimpanzees living in larger groups can arguably be said to lead richer lives. Specifically, when compared to their conspecifics residing in pairs or trios, they engage in more frequent play and grooming, and are responsible for initiating more of these interactions. They have lower agonistic display rates when compared to those in pairs or trios, whose actions could be taken as a sign of frustration or boredom. Although they spend less time alone, they also have lower median association times and fewer significant partners. This reflects the opportunity for those in larger groups to distribute interactions across a higher number of other partners rather than having to spend disproportionate amounts of time with only one or two others. Analyses of variance on the personality ratings of these chimpanzees have already identified group size as influencing ratings on adjectives such as ‘Sociable’ and ‘Popular’ [22]. Here, group size is found to affect median association time, a measure which could be predicted, somewhat counter-intuitively, to correlate inversely with ‘Sociable’ and ‘Popular’ ratings. That is, individuals with lower association times (because of their larger groupings) spread themselves among, or are sought out by, a greater number of partners. Therefore, ‘association’ cannot be said to measure systematically or necessarily ‘sociability’ per se, as is commonly held; see, e.g., [13]. A more sociable ape tends to spend less time with each partner, but only because there are more partners available. Totaling time spent in association regardless of with which particular partner, and comparing that with the amount of time spent alone could, however, yield a broader measure of sociability. Even this is problematic, however, in the sense that ‘sociable’ chimpanzees were not found to spend significantly less time alone than less sociable ones, even though chimpanzees rated higher on the term ‘solitary’ do spend more time alone [16].

By examining the top three partners with whom each subject spent the greatest amount of time there is evidence that, within a larger group, individuals tend to cluster [44]. Within the group at Zoo A, for example, two principal clusters of associating individuals existed. One cluster comprised purely adults, being the four adult males along with four adult females, two of whom had daughters between four and five years old. The second cluster of individuals was made up of the related mothers and their mainly ventral infants, together with their older offspring. Associating with individuals in both of these clusters were three ‘floating’ immatures. They were attached to their mothers in the first cluster and to other favored partners, mainly other immatures, in the second cluster. A final trio of ‘floaters’ existed, comprising the other mother with her ventral infant and the two eldest females.

Chimpanzees in smaller groups (but still ≥ seven) at Zoos B and C, however, had more evenly distributed association patterns among group members. Those individuals housed in pairs and trios obviously had very different association patterns. Favorite partners frequently existed and, consequently in trios, another individual became an ‘outsider’. It is notable that one third of the nine chimpanzees housed in trios at Zoo C were never seen to engage in grooming activity, making them the only chimpanzees not to groom at all when at least two other partners were available. By contrast, chimpanzees in larger groups were much more likely to spend greater amounts of time engaged in grooming. Reciprocity in grooming can be seen to involve four directions: A grooms B, B grooms A, A is groomed by B, and B is groomed by A. Totally reciprocal grooming relationships (regarding top partners) often involve related individuals, particularly mothers and their offspring, which concurs with findings in wild apes [13,45].

Agonistic displays are a prominent feature in the lives of chimpanzees. What elicits the display can be hard to determine but it is largely driven by status acquisition and reinforcement [46,47]. While such displays and acts of overt aggression are often directed at one or more specific others, they are frequently general, being directed at no obvious target. Frustration, boredom, monotony, lack of variety and the anthropomorphic ‘clash of personalities’ are likely to be major factors contributing to the aggressive and display behavior of those chimpanzees housed in trios and pairs in neighboring enclosures at Zoo C. These males and certain females had relatively high display rates, often directed at other males and females able to be seen, but not physically contacted. While the presence of several males in a captive group of chimpanzees may facilitate the expression of species-typical behaviors such as displays, the present findings do not point to a significant difference between the display rates of the dominant male in the multi-male group and the single male in the other large group. Other studies have found lower rates in single males, and attribute this to a lack of motivation for assertion [48]. However, the presence of several males is likely to provide more choice of preferred social partners for these males, and is a more natural social context.

The relative lack of play among the chimpanzees at Zoo C is striking compared to the play patterns of the individuals at the other zoos. That this lack or indeed absence of play is related to being housed in small groups and likely having experienced hand-rearing confirms previous findings of the importance of the mother’s influence on the expression of social behaviors [34] and specifically, in fostering play while young [13,35]. Immatures are arguably the most popular play partners of both adults and other immatures to the extent that, without the presence of youngsters to provide the impetus for play, adult chimpanzees grouped together did not engage in this activity at all. This concurs with Bloomsmith’s [49] finding that captive adult male chimpanzees are attracted to immatures, and they may be more likely to develop affiliative relationships with infants and juveniles because of an increase in the social complexity of captive group life. It provides some evidence for the much-debated suggestion that youngsters are essential in chimpanzee groups to optimize welfare; however, see [50]. Indeed, building on a recent suggestion that play may be a luxury that nonhuman animals can only afford in times of high-quality food abundance and that engaging in play may be a necessary ‘hidden cost’ for wild chimpanzee mothers [51], it could be expected that captive chimpanzees would engage in more consistent amounts of play due to their lack of uncertainty over energy expenditure in foraging.

Such findings therefore have important implications for the housing of apes in captivity. It is also worth noting the importance of the choice of measure. Here, both frequencies and durations were recorded. Median grooming was higher in small groups while rate of grooming was higher in large groups. This can be explained in terms of the number of available partners in the group but it also illustrates how the decision to use just one of these measures could impact and bias the results of a study. This consideration may not necessarily apply to all forms of social behavior. For example, both median and rate of play were higher in large than small groups. A limitation of this study is that other factors could potentially influence social behavior; for example, differences in the size and contents of the enclosures and differences in husbandry. However, the findings are important in helping zoo managers and keepers maximize the welfare of captive chimpanzees by emphasizing the need to take into account the importance of chimpanzees’ early experience and how that can act alone, and in combination with other factors, including age and sex, and the size of the group in which they live, in determining their trajectory within a captive group. 

Being housed with conspecifics from all age-sex classes not only enables individuals to learn from each other, but permits choice of preferred social partners. In situations where, for some reason, chimpanzees are kept in pairs or trios, it is vital that the individuals are chosen carefully. They will likely (be forced to) spend disproportionately large amounts of time with only one or two other conspecifics. It need not be that the chimpanzees are wholly similar in terms of their personality [22] or behavior, because matching a mother-reared chimpanzee with a hand-reared one could facilitate a bond due to the former being more likely to take more responsibility to solicit grooming. That mother-reared chimpanzees have the social competence needed to approach others and solicit grooming is an important finding. Arguably, most animal mothers would wish their offspring to be competent and confident in life and, indeed, I have shown here that hand-reared individuals likely lack this confidence as they do not take the responsibility to ask for grooming from their conspecifics. It is akin to the shy child sitting on the friendship bench waiting to be approached. On the other hand, both mother-reared and hand-reared chimpanzees perform agonistic displays more frequently when housed in a smaller group (compared to large), and so attention needs to be paid to the wider surroundings of chimpanzees housed in pairs or trios, as displaying due to the presence of nearby but out-of-reach conspecifics could be mitigated. This supports other calls for zoos to consider issues such as allowing sufficient physical space per individual to mitigate potential aggression [10].

## 5. Conclusions

In conclusion, these findings support the importance of early life experiences and current social context in shaping social competence [23,24]. Chimpanzees reared by their mothers have the social competence needed to take responsibility for approaching others to be groomed. When they live in groups of at least seven, as opposed to in pairs or trios, they have lower median association times and fewer partners with whom they spend a disproportionate amount of time, instead dispersing their affiliative interactions among group members. They spend less time alone, perform fewer agonistic displays and engage in both more play and more grooming. These findings affirm the intuitive belief that, certainly in a species such as the chimpanzee, the optimum captive environment should be based on large social groupings of individuals from all age and sex classes. 

## Figures and Tables

**Table 1:** Group composition at Zoos A, B and C.

Grouping	Group Size	Sexes	Ages	Rearing	Dominance
**Zoo A**					
Group	22	M 6, F 16	<2–45	H 11, M 11	1 male
**Zoo B**					
Group	11	M 4, F 7	<2–26	H 4, M 7	1 male
**Zoo C**					
Group	7	M 2, F 5	9–35	H 5, M 2	1 male
Trio	3	M 1, F 2	16–23	H 3, M 0	1 male
Trio	3	M 2, F 1	16–34	H 2, M 1	1 male
Trio	3	M 1, F 2	13–21	H 3, M 0	1 male
Pair	2	M 1, F 1	18, 27	H 1, M 1	1 male
Pair	2	M 1, F 1	10, 14	H 2, M 0	1 male

**Table 2:** Distribution of subjects by grouping, rearing and age categories.

Grouping	Large Group (≥7)		Small Group (≤3)	
Rearing	Hand-Reared	Mother-Reared	Hand-Reared	Mother-Reared
Age				
Young Adults (9–20 y)	12	5	7	2
Mature Adults (≥21 y)	9	0	4	0
Total	21	5	11	2

**Table 3:** Behavioral ethogram and indexes.

Behavior	Definition	Behavior Index	Definition
Time in Proximity	Partners within two arms’ reach	Median Association Time	Time A and B spent together (within two arms’ reach) divided by total time A and B were available to spend time together
Time Alone	No partners within two arms’ reach	% Time Spent Alone	Time spent alone divided by total observation time
Approaches/Leaves another	Identify who approaches and who leaves who	Responsibility for Proximity	Calculated using the Hinde and Atkinson [41] formula which counts the number of approaches and leaves made by members of a dyad
Proximity Partners	Identify proximity partners	% Significant Partners	Partners each subject associated with for percentages of time ≥ than sample median
Groom	Fingers or mouth picking through fur	Rate of Grooming	Time spent grooming divided by total observation time
Groom Received	Another picks/mouths focal’s fur	Rate Being Groomed	Time spent being groomed divided by total observation time
Groomer Partners	Identify grooming partners	% Significant Groomers	Partners each subject was groomed by for percentages of time ≥ than sample median
Groomed Partners	Identify groomed partners	% Significant Partners Groomed	Partners each subject groomed for percentages of time ≥ than sample median
Initiates/Terminates groom	Identify who initiates and who terminates request	Responsibility as Groomee	Composite score illustrating responsibility as groomee with significant partners calculated using the Hinde and Atkinson [41] formula
Initiates/Terminates groom another	Identify who initiates and who terminates request	Responsibility as Groomer	Composite score illustrating responsibility as groomer with significant partners calculated using the Hinde and Atkinson [41] formula
Aggression	Hits, kicks, pushes another	Rate of Aggression	Sum of aggressive incidents divided by total observation time
Agonistic display	Charges, slaps, bristles, sways, bluffs, usually accompanied by vocalization	Rate of Display	Sum of displays divided by total observation time
Play	Positive engagement with another in active manner, possibly with play face, laughter	Rate of Play	Time spent playing divided by total observation time
Play Partners	Identify play partners	% Significant Play Partners	Partners each subject played with for percentages of time ≥ than sample median
Initiates/Terminates play with another	Identify who initiates and who terminates play	Responsibility for Play	Composite score illustrating responsibility for play with significant partners calculated using the Hinde and Atkinson [41] formula

**Table 4:** PCA loadings of the behavior codings at T1 on Components 1 to 5.

Component	Grooming	Play	Sociability	Aggression	Responsibility
% Variance Explained	18.5%	16.8%	16.0%	12.3%	10.5%
Rate of Grooming	**0.889**	−0.114	0.010	−0.013	0.062
Rate Being Groomed	**0.872**	−0.045	−0.088	−0.147	−0.090
Rate of Play	−0.194	**0.838**	−0.229	−0.117	0.170
% Significant Play Partners	−0.315	**0.825**	0.040	0.303	0.067
% Time Alone	−0.382	**−0.779**	−0.004	0.171	0.237
% Significant Partners	−0.218	0.170	**0.826**	0.063	−0.156
Median Association Time	**−0.562**	−0.006	**0.682**	0.151	−0.192
% Significant Partners Groomed	0.339	−0.225	**0.663**	0.021	0.149
% Significant Groomers	0.256	−0.172	**0.657**	**−0.516**	−0.118
Rate of Agonistic Display	−0.289	−0.197	**0.520**	**0.411**	−0.053
Rate of Aggression	−0.191	0.218	0.183	**0.741**	−0.114
Responsibility as Groomee	−0.149	0.342	0.083	**−0.699**	0.304
Responsibility for Proximity	−0.205	−0.082	−0.112	−0.073	**0.828**
Responsibility as Groomer	0.199	0.093	−0.051	−0.252	**0.599**
Responsibility for Play	**0.418**	**0.430**	−0.002	0.347	**0.480**

Note. A sample size of 59 was used to calculate the correlation matrix. Values in bold indicate an adequate (≥|0.4|) loading.

**Table 5:** Mean (SD) scores and measures for chimpanzees on behavior components and behavior codings by grouping and rearing.

Measure	Large Group (*N* = 26)		Small Group (*N* = 13)	
	Mother-Reared	Hand-Reared	Mother-Reared	Hand-Reared
	(*N* = 5)	(*N* = 21)	(*N* = 2)	(*N* = 11)
*Principal components*				
Grooming	0.76 (0.96)	0.66 (1.06)	−0.38 (0.26)	−0.64 (0.59)
Play	0.06 (0.78)	−0.50 (0.62)	−1.15 (0.30)	−0.89 (0.45)
Sociability	−0.16 (0.76)	−0.45 (0.64)	1.66 (1.72)	0.82 (1.24)
Aggression	−0.02 (0.55)	−0.00 (0.51)	−0.46 (1.21)	−0.25 (0.86)
Responsibility	−0.03 (0.67)	−0.16 (1.01)	1.20 (1.53)	−0.30 (1.06)
*Behavior codings*				
% Time alone	16.22 (16.32)	32.86 (16.56)	51.95 (11.81)	40.76 (15.88)
Median association time	13.06 (5.49)	12.87 (8.23)	38.85 (1.20)	43.67 (9.95)
% Significant partners	57.80 (18.29)	40.571(15.95)	75.00 (35.36)	85.36 (23.35)
Responsibility for proximity	0.55 (0.30)	0.47 (0.47)	1.000 (1.41)	0.59 (0.49)
Median time grooming	23.50 (9.62)	31.19 (17.56)	71.25 (51.27)	51.82 (58.83)
Median time being groomed	21.00 (10.84)	27.98 (10.97)	105.00 (113.14)	55.46 (61.85)
% Significant partners groomed	40.40 (13.20)	37.38 (19.16)	75.00 (35.36)	54.55 (41.56)
% Significant groomers	35.80 (22.52)	41.48 (18.15)	75.00 (35.36)	54.55 (41.56)
Rate giving grooming	15.05 (8.09)	13.55 (8.77)	10.98 (7.89)	4.06 (4.33)
Rate receiving grooming	11.40 (7.28)	15.62 (8.94)	8.79 (7.68)	4.46 (4.56)
Responsibility as groomer	1.00 (0.21)	0.92 (0.85)	1.50 (0.71)	0.50 (1.20)
Responsibility as groomee	0.67 (0.39)	0.57 (0.50)	1.50 (0.71)	0.50 (0.50)
Median play duration	16.50 (11.12)	14.52 (13.78)	0.00 (0.00)	2.73 (4.67)
% Significant play partners	12.60 (13.45)	10.43 (11.57)	0.00 (0.00)	0.00 (0.00)
Responsibility for play	0.79 (1.02)	0.51 (0.71)	0.00 (0.00)	0.00 (0.00)
Rate of play	5.52 (4.19)	3.71 (4.67)	0.00 (0.00)	0.28 (0.53)
Rate of aggression	0.11 (0.13)	0.12 (0.11)	0.27 (0.27)	0.06 (0.16)
Rate of agonistic display	0.06 (0.07)	0.32 (0.50)	3.62 (5.11)	1.24 (1.15)

**Table 6:** Model results from mixed-effect linear regressions predicting the influence of rearing and grouping on Grooming scores.

Predictors	*β* Estimate	SE	95% CI (LL, UL)	*t*	*p*
**Fixed effects**					
Intercept	−0.64	0.28	−1.21, −0.08	−2.31	**0.027**
Rearing	0.27	0.71	−1.17, 1.70	0.38	0.708
Grouping	1.30	0.34	0.60, 1.99	3.80	**<0.001**
**Random effects**	*β* Estimate	*Wald Z*	*p*		
Subject ID	0.28	1.55	0.122		

Note. *N* = 39. CI: confidence interval, lower limit, upper limit; *p* values < 0.05 are shown in bold; AIC = 107.211.

**Table 7:** Model results from mixed-effect linear regressions predicting the influence of grouping on measures of social competence.

Predictors	*β* Estimate	SE	95% CI (LL, UL)	*t*	*p*
**Fixed effects**					
% Time alone					
Intercept	40.76	4.89	30.83, 50.69	8.33	**<0.001**
Grouping	−7.90	6.04	−20.16, 4.36	−1.31	0.199
Median association time					
Intercept	43.66	2.53	38.51, 48.80	17.24	**<0.001**
Grouping	−30.79	3.13	−37.13, −24.44	−9.85	**<0.001**
% Significant partners					
Intercept	86.36	5.84	74.51, 98.22	14.79	**<0.001**
Grouping	−45.79	7.21	−60.43, −31.16	−6.35	**<0.001**
Median grooming time					
Intercept	51.82	10.66	30.17, 73.47	4.86	**<0.001**
Grouping	−20.63	13.16	−47.35, 6.10	−1.57	0.126
Significant partners groomed					
Intercept	54.55	8.31	37.68, 71.41	6.57	**<0.001**
Grouping	−17.17	10.25	−37.98, 3.65	−1.67	0.103
Median time being groomed					
Intercept	55.46	11.84	31.43, 79.48	4.69	**<0.001**
Grouping	−27.48	14.61	−57.14, 2.18	−1.88	0.068
% Significant groomers					
Intercept	54.55	8.40	37.50, 71.59	6.50	**<0.001**
Grouping	−13.07	10.36	−34.11, 7.97	−1.26	0.216
Rate of Grooming					
Intercept	4.06	2.31	−0.62, 8.75	1.76	0.087
Grouping	9.49	2.85	3.71, 15.27	3.33	**0.002**
Rate being groomed					
Intercept	4.46	2.32	−0.25, 9.18	1.92	0.063
Grouping	11.16	2.87	5.34, 16.98	3.89	**<0.001**
Agonistic display rate					
Intercept	1.24	0.34	0.55, 1.93	3.66	**<0.001**
Grouping	−0.93	0.42	−1.78, −0.08	−2.21	**0.034**
Rearing * Grouping	−2.63	1.03	−4.72, −0.54	−2.55	0.015
Median play duration					
Intercept	2.73	3.42	−4.22, 9.67	0.80	0.431
Grouping	11.80	4.22	3.22, 20.37	2.79	**0.008**
Rate of play					
Intercept	0.28	1.15	−2.05, 2.62	0.25	0.807
Grouping	3.42	1.42	0.54, 6.30	2.41	**0.021**
% Significant play partners					
Intercept	−6.94 (E-16)	2.97	−6.03, 6.03	0.00	1.00
Grouping	10.43	3.67	2.98, 17.88	2.84	**0.007**
Responsibility for play					
Intercept	3.47 (E-17)	0.19	−0.39, 0.39	0.00	1.00
Grouping	0.51	0.24	0.03, 0.99	2.16	**0.038**
**Random effects**	*β* Estimate	*Wald Z*	*p*		
% Time alone					
Subject ID	87.74	1.55	0.122		
Median association time					
Subject ID	23.51	15.21	0.122		
% Significant partners					
Subject ID	125.05	80.91	0.122		
Median grooming time					
Subject ID	416.97	269.78	0.122		
Significant partners groomed					
Subject ID	253.00	1.55	0.122		
Median time being groomed					
Subject ID	513.70	1.55	0.122		
% Significant groomers					
Subject ID	258.46	1.55	0.122		
Rate of Grooming					
Subject ID	19.52	1.55	0.122		
Rate being groomed					
Subject ID	19.78	1.55	0.122		
Agonistic display rate					
Subject ID	0.422	1.55	0.122		
Median play duration					
Subject ID	42.94	1.55	0.122		
Rate of play					
Subject ID	4.85	1.55	0.122		
% Significant play partners					
Subject ID	32.38	1.55	0.122		
Responsibility for play					
Subject ID	0.14	1.55	0.122		

Note. *N* = 39. CI: confidence interval, lower limit, upper limit; only significant interactions are shown; *p* values < 0.05 are shown in bold; * shows interactions; AIC = 64.216 to 369.979.

**Table 8:** Model results from mixed-effect linear regressions predicting the influence of rearing and grouping on Play scores.

Predictors	*β* Estimate	SE	95% CI (LL, UL)	*t*	*p*
**Fixed effects**					
Intercept	−0.89	0.18	−1.25, −0.53	−5.00	**<0.001**
Rearing	−0.26	0.45	−1.18, 0.66	−0.58	0.568
Grouping	0.39	0.22	−0.06, 0.83	1.77	0.085
**Random effects**	*β* Estimate	*Wald Z*	*p*		
Subject ID	0.12	1.55	0.122		

Note. *N* = 39. CI: confidence interval, lower limit, upper limit; *p* values < 0.05 are shown in bold; AIC = 76.069.

**Table 9:** Model results from mixed-effect linear regressions predicting the influence of rearing and grouping on Sociability scores.

Predictors	*β* Estimate	SE	95% CI (LL, UL)	*t*	*p*
**Fixed effects**					
Intercept	0.82	0.27	0.27, 1.37	3.00	**0.005**
Rearing	0.84	0.70	−0.57, 2.25	1.21	0.235
Grouping	−1.27	0.34	−1.96, −0.59	−3.78	**<0.001**
**Random effects**	*β* Estimate	*Wald Z*	*p*		
Subject ID	0.27	1.77	0.122		

Note. *N* = 39. CI: confidence interval, lower limit, upper limit; *p* values < 0.05 are shown in bold; AIC = 106.063.

**Table 10:** Model results from mixed-effect linear regressions predicting the influence of rearing and grouping on Aggression scores.

Predictors	*β* Estimate	SE	95% CI (LL, UL)	*t*	*p*
**Fixed effects**					
Intercept	−0.25	0.2	−0.65, 0.15	−1.26	0.216
Rearing	−0.22	0.51	−1.24, 0.81	−0.43	0.673
Grouping	0.25	0.25	−0.25, 0.75	1.02	0.315
**Random effects**	*β* Estimate	*Wald Z*	*p*		
Subject ID	0.14	1.55	0.122		

Note. *N* = 39. CI: confidence interval, lower limit, upper limit; AIC = 83.672.

**Table 11:** Model results from mixed-effect linear regressions predicting the influence of rearing on measures of social competence.

Predictors	*β* Estimate	SE	95% CI (LL, UL)	*t*	*p*
**Fixed effects**					
Responsibility as groomee					
Intercept	0.5	0.15	0.20, 0.81	3.33	**0.002**
Rearing	1.00	0.38	0.22, 1.78	2.61	**0.013**
Agonistic display rate					
Intercept	1.24	0.34	0.55, 1.93	3.66	**<0.001**
Rearing	2.37	0.87	0.62, 4.13	2.75	**0.009**
Rearing * Grouping	−2.63	1.03	−4.72, −0.54	−2.55	**0.015**
**Random effects**	*β* Estimate	*Wald Z*	*p*		
Responsibility as groomee					
Subject ID	0.08	1.55	0.122		
Display rate					
Subject ID	0.422	1.55	0.122		

Note. *N* = 39. CI: confidence interval, lower limit, upper limit; only significant interactions are shown; *p* values < 0.05 are shown in bold; * shows interactions; Akaike’s Information Criterion (AIC) = 64.216–121.301.

**Table 12:** Model results from mixed-effect linear regressions predicting the influence of rearing and grouping on Responsibility scores.

Predictors	*β* Estimate	SE	95% CI (LL, UL)	*t*	*p*
**Fixed effects**					
Intercept	−0.30	0.30	−0.92, 0.32	−0.99	0.331
Rearing	1.50	0.78	−0.07, 3.08	1.94	0.061
Grouping	0.14	0.38	−0.62, 0.91	0.38	0.707
**Random effects**	*β* Estimate	*Wald Z*	*p*		
Subject ID	0.34	1.55	0.122		

Note. *N* = 39. CI: confidence interval, lower limit, upper limit; AIC = 113.743.

## Data Availability

Data are available on the Open Science Framework at https://osf.io/sxjza (accessed on 12 January 2025).
